# Acceptability of a digital health intervention alongside physiotherapy to support patients following anterior cruciate ligament reconstruction

**DOI:** 10.1186/s12891-017-1846-0

**Published:** 2017-11-21

**Authors:** Emma Dunphy, Fiona L. Hamilton, Irena Spasić, Kate Button

**Affiliations:** 1 E-Health Unit, Research Department of Primary Care and Population Health, Upper Third Floor UCL Medical School (Royal Free Campus), Rowland Hill Street, NW3 2PF, London, UK; 2 Homerton University Hospital NHS Trust, Homerton Row E96SR, London, UK; 3 School of Computer Science & Informatics, Cardiff University, Queens Building, 5 The Parade, Cardiff, CF24 3AA UK; 4 School of Healthcare Sciences, Cardiff University, Eastgate House, Newport Road, Cardiff, CF24 0AB UK; 5 Cardiff & Vale University Health Board, Health Park, Cardiff, CF14 4XW UK

**Keywords:** Anterior cruciate ligament, Physiotherapy, Surgery, Rehabilitation, E-health, Internet

## Abstract

**Background:**

Physiotherapy rehabilitation following surgical reconstruction to the Anterior Cruciate Ligament (ACL) can take up to 12 months to complete. Given the lengthy rehabilitation process, a blended intervention can be used to compliment face-to-face physiotherapy with a digital exercise intervention. In this study, we used TRAK, a web–based tool that has been developed to support knee rehabilitation, which provides individually tailored exercise programs with videos, instructions and progress logs for each exercise, relevant health information and a contact option that allows a patient to email a physiotherapist for additional support. The aim of this study was to evaluate the acceptability of TRAK–based blended intervention in post ACL reconstruction rehabilitation.

**Methods:**

A qualitative research design using semi-structured interviews was used on a convenience sample of participants following an ACL reconstruction, and their treating physiotherapists, in a London NHS hospital. Participants were asked to use TRAK alongside face-to-face physiotherapy for 16 weeks. Interviews were carried out, audio recorded, transcribed verbatim and coded by two researchers independently. Data were analyzed using thematic analysis.

**Results:**

Of the 25 individuals that were approached to be part of the study, 24 consented, comprising 8 females and 16 males, mean age 30 years. 17 individuals used TRAK for 16 weeks and were available for interview. Four physiotherapists were also interviewed. The six main themes identified from patients were: the experience of TRAK rehabilitation, personal characteristics for engagement, strengths and weaknesses of the intervention, TRAK in the future and attitudes to digital healthcare. The main themes from the physiotherapist interviews were: potential benefits, availability of resources and service organization to support use of TRAK.

**Conclusions:**

TRAK was found to be an acceptable method of delivering ACL rehabilitation alongside face-to-face physiotherapy. Patients reported that TRAK, specifically the videos, increased their confidence and motivation with their rehabilitation. They identified ways in which TRAK could be developed in the future to meet technological expectations and further support rehabilitation. For Physiotherapists time and availability of computers affected acceptability. Organization of care to support integration of digital exercise interventions such as TRAK into a blended approach to rehabilitation is required.

## Background

Anterior cruciate ligament (ACL) injury represents a significant burden of serious knee injuries. An American study estimates that 100,000 ACL reconstructions are performed per year [1]. A UK based study estimates that in a ‘catchment area of 400,000 population, accident and emergency departments can expect to see two acute ACL injuries per week [2]. There is a significant commitment to rehabilitation required for patients who wish to return to an active, athletic life [3–5].

**Fig. 1:**
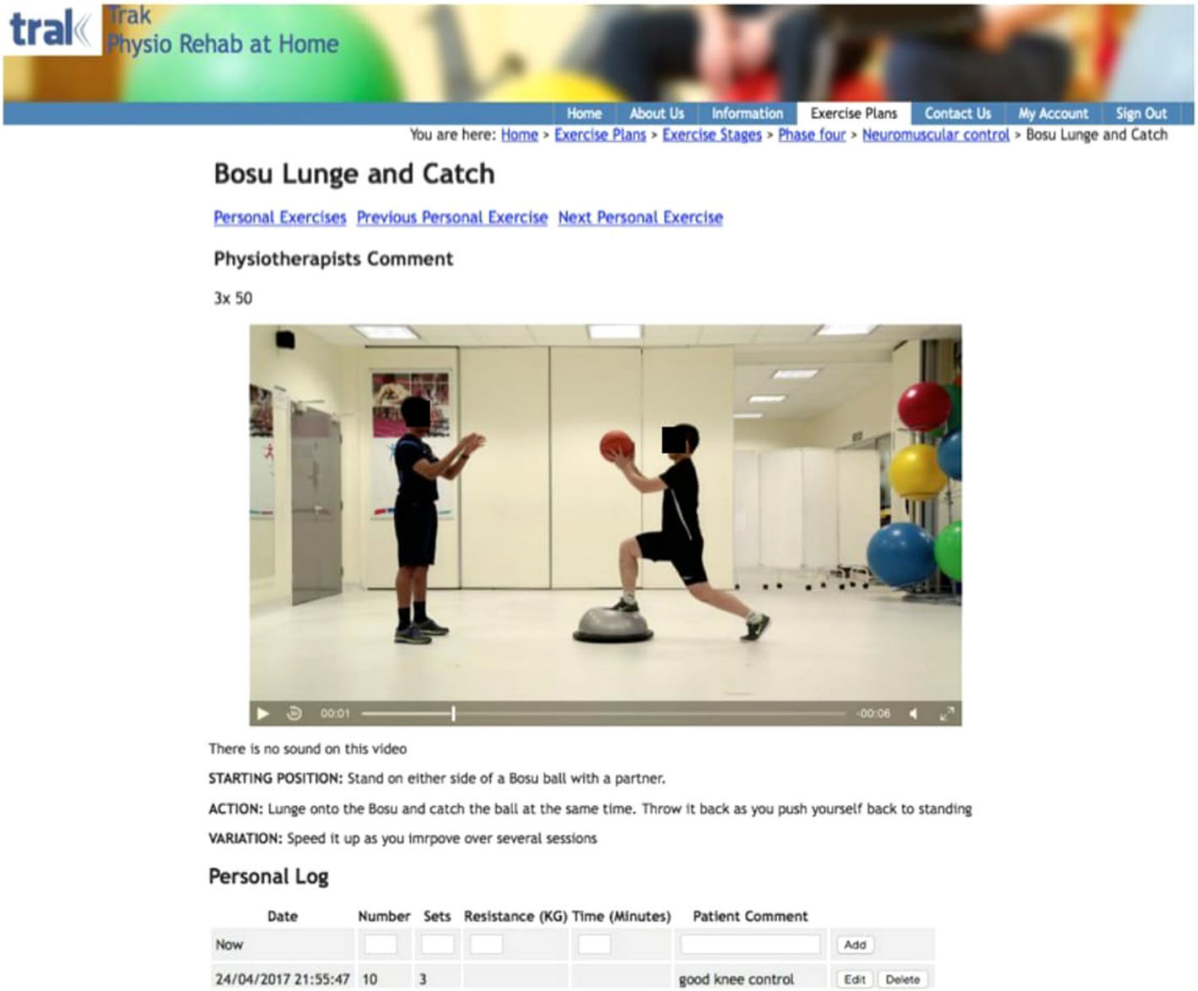
A description of a neuromuscular control exercise in Phase 4 of rehabilitation where the goal is return to sport activities. (From TRAK website with permission)

There are many protocols for ACL rehabilitation available and evidence for both physical and psychological recovery continues to inform best practice in optimizing function [5–10]. Still, a high percentage of individuals never return to their pre-injury level of function [3, 6, 11–14]. Potential reasons for this are loss of confidence and self-efficacy [6, 11, 15] combined with poor engagement with the rehabilitation process [16], which is known to be lengthy in nature, often requiring commitment for a year or more [14]. Patients struggle with motivation, compliance and a clear understanding of what is recommended at each stage of their recovery [5, 16]. Patient knowledge and engagement are key to successful rehabilitation [17]. There is evidence to suggest that digital tools can support engagement with healthcare [18–22]. Patients can use the Internet to gain access to correct information and utilize digital tools such as progress logs and personalized prompts in order to be informed, motivated and encouraged [21, 23, 24]. One such tool is TRAK, a digital intervention developed to support self-management of knee conditions [24, 25]. TRAK, provides a platform for individually tailored exercise programs with videos, detailed instructions and progress logs for individual exercises, a health information section, and a contact option that allows a patient to email a physiotherapist for additional support. TRAK has recently been modified with specific content for ACL rehabilitation based on the best available evidence [4, 5, 9, 10, 16, 26]. The ACL-specific version of TRAK includes over 200 new exercise videos, mostly to support advanced stages of physiotherapy including return to sport activities. These exercises (see Fig. 1 for an example), target modifiable factors such as strength, neuromuscular control, motor learning, sport specific skills and fatigue resistance, and are specifically tailored to an ACL population of patients, who tend to be younger and more active, and therefore have higher expectations regarding knee function [27]. ACL-related health information focuses on managing expectations given a lengthy recovery process by providing detailed information, milestones and common problems associated with each stage of rehabilitation.

As part of the development and testing of digital interventions, we need to understand the relationship between individuals’ initial reactions to newly developed online interventions, the intention to use it and their actual use [28]. The usability study conducted for the initial version of TRAK, which was implemented originally as a Facebook app, suggested that a digital exercise intervention would facilitate communication, provide information, help recall information, improve understanding, enable exercise progression, and support self-management in general [24]. These finding were subsequently confirmed when a new version of TRAK re–implemented as web–based app (which is also the version discussed in this study) was integrated into routine healthcare and evaluated for its impact on the patient, clinician and organization [25, 29]. The aim of the current study, however, was to evaluate the acceptability of TRAK to a specific patient population – those following ACL reconstruction, whose rehabilitation on average takes 12 months to complete and, therefore, may struggle with long–term engagement. In addition to patients themselves, we also evaluated the acceptability of TRAK to physiotherapists. These findings will be used to inform more effective integration of TRAK into a blended exercise intervention, which should capture patients’ needs over an extended duration of recovery pathway.

## Methods

### Patient public involvement

Patients and the public were involved in the early development of this study by suggesting exercise content appropriate to their rehabilitation goals. They participated in the filming of videos and gave consent for public use of these videos. They informed the study methodology by suggesting that a trial period of usage who be suitable to assess the website acceptability. This was not the group of patients who were the recruited to the study.

### Study design

This research was done with qualitative methods using semi-structured interviews to ascertain the acceptability of TRAK to a population receiving rehabilitation following ACL reconstruction as well as their treating physiotherapists, who were based at one London NHS hospital. Five physiotherapists were trained on how to use TRAK and integrate it into patient care alongside face-to-face physiotherapy. They then used TRAK for four months with a group of patients who were at varying stages following ACL reconstruction. After four months semi-structured interviews were conducted by lead researcher ED, at a location convenient to both types of participants. The interviews focused on the overall experience of ACL rehabilitation and in particular experience of using TRAK. In relation to the latter, we examined the influence of contextual factors related to acceptability of new digital tools including performance expectancy, effort expectancy, social influence, facilitating conditions, self-efficacy, anxiety and behavioral intentions [28].

### Standard ACL rehabilitation pathway

All patients who undergo ACL reconstruction are invited to follow a standard ACL rehabilitation pathway, which has been designed around published evidence and is delivered in a group format. [5, 7–10, 16, 26, 30]. This pathway consists of four rehabilitation stages (Table 1.). Progression through the stages is based on pre-set functional criteria. Treatment is delivered within a group environment. Individual consultations are provided for within that structure. Exercise prescription is individually tailored and in line with American College of Sports Medicine guidance [31].

**Table 1:** Stages of ACL rehabilitation in standard care

Stage 1, 0–6 weeks approximately, depending on milestones achieved: focuses on restoration of active range of motion, muscle activation, gait and the management of swelling, pain and wound healing.
Stage 2, 6 weeks to 3 months approximately, depending on milestones achieved: weekly classes which initiate strength training and neuromuscular control.
Stage 3, from 3 months approximately, depending on milestones achieved: strength gains are consolidated and motor control is challenged with increasing dynamic activity. Some participants will complete care and return to sport in this stage where goals do not include cutting and pivoting activities such football, hockey, dancing etc.
Stage 4, from 6 months approximately, depending on milestones achieved: advanced sports specific skills and movement patterns are trained toward patient goals.

There is a significant variety in patients’ individual experiences and goals. TRAK rehabilitation is designed so that it can reflect any individual’s pathway within these stages. It is designed to facilitate improved engagement, confidence, motivation and self-efficacy away from the class. The personalized exercise plans with videos, exercise log, information and remote e-mail support for any questions or concerns are in addition to the standard care ACL program.

### Sample selection and recruitment

**Fig. 2:**
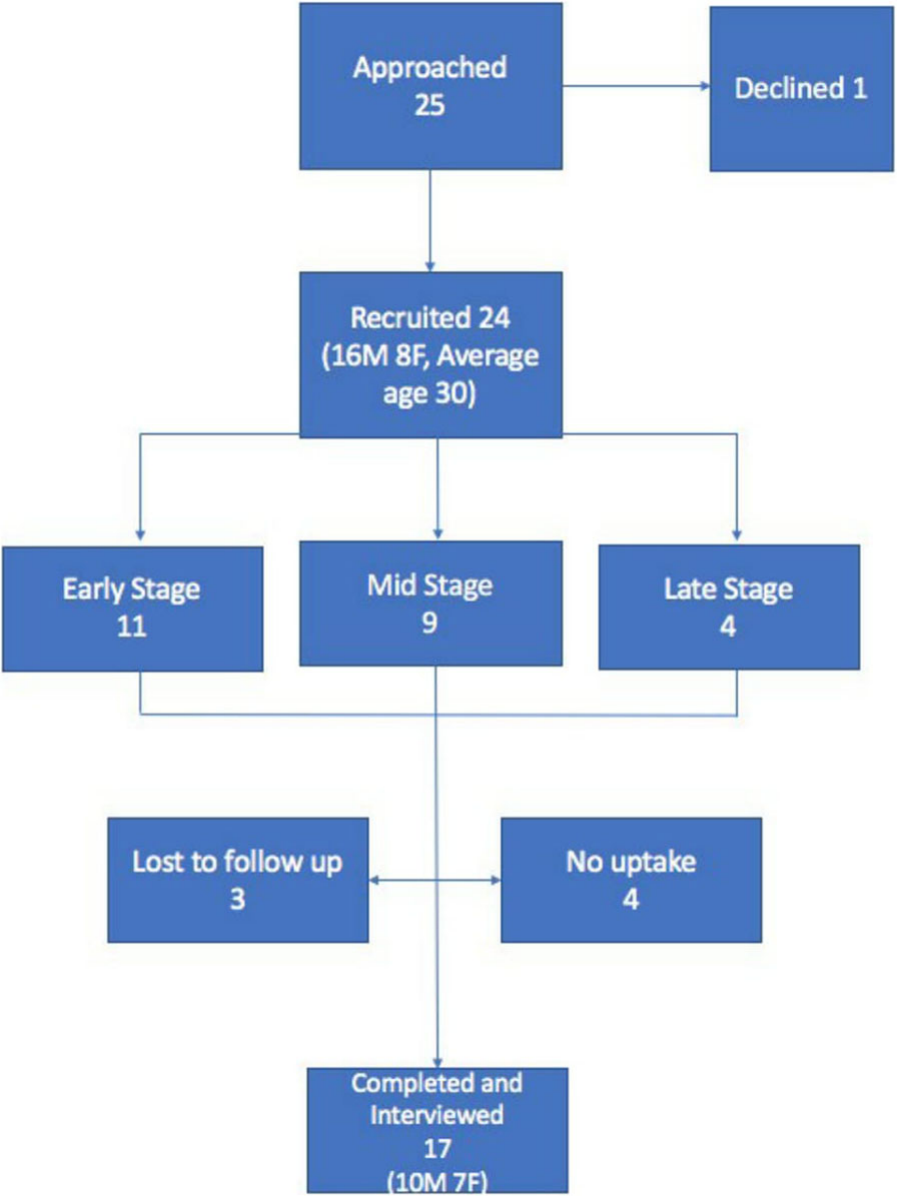
Sample and Recruitment

This study took place in a London hospital with an NHS caseload of predominantly recreational athletes. All patients were recruited from the physiotherapy department, where they were receiving treatment following ACL reconstructive surgery. Between two and four new ACL patients are referred weekly from orthopedics. A convenience sample method was used: patients were recruited from all stages of the physiotherapy post-operative ACL pathway (see Fig. 2). The inclusion criteria were: all patients on the ACL pathway who could freely consent, were over 18 years old, who spoke English fluently and had access to a smartphone. The exclusion criteria were: patients with multi-ligament reconstruction, revision ACL surgery or fractures, or patients with a poor command of English. Twenty-five patients were asked to take part in the study. One patient declined participation because of time concerns. A sample of 24 patients was recruited. Four patients withdrew from the study due to time constraints. Seventeen patients completed the study and were interviewed. Following provision of an information leaflet and obtaining written informed consent, participants were trained on how to use TRAK by their treating physiotherapist who provided them with a personalized exercise plan. This was delivered in a one-to-one session and patients were offered informal user guidance as needed.

The Principal Investigator (PI) was one of 5 treating physiotherapists in the ACL group from Band 5, 6 and 7 level of experience. Thirteen participants knew the PI before being recruited but were unaware of any role or motivation in the study. Physiotherapist training on how to use TRAK as part of the standard care took place over a 30 min period in a group session led by the lead researcher. The TRAK exercise programs were maintained and updated by all physiotherapists involved in the group.

### Data collection

Semi-structured interviews took place at a convenient time and place for the participants, at the hospital, over the phone and in two cases at an agreed, appropriate public place. All physiotherapists were interviewed in the physiotherapy department. All interviews were digitally audio recorded and transcribed verbatim by the lead researcher. All interviews were participant and PI only.

The interview questions were guided by topics that arose in previous related studies and from ongoing informed discussion within the research team [20, 24, 25]. The topic guide was minimally refined throughout the process to responds to emerging data.

### Data analysis

Data from the interviews were analyzed using pragmatic thematic analysis to inform factors that relate to acceptability of new digital tools like TRAK. This sees the data itself rather than the theory driving the process. The themes emerging from the interviews are grouped stringently so all the interview data relating to a particular theme are recorded. Data are weighted and reported secondary to frequency of occurrence or explanatory value [32]. The data were analyzed and coded by the principal investigator and separately coded by another team member. Saturation of data was agreed upon. A dialectic process followed until agreement was reached. Data are presented in the results as a descriptive narrative to reflect the patient and physiotherapist experience [32].

## Results

Ten male and seven female patients with an average age of 30 were interviewed. Four Physiotherapists were also interviewed. The patient and physiotherapists interviews have been reported separately to illustrate with clarity how using TRAK alongside face-to-face physiotherapy was experienced by patients and physiotherapists. Key quotes from the interviews are included to express the specific words of the participants. The results show how and why users accept or do not accept TRAK. They explore in particular, performance and effort expectancy, social conditions and environment, self-efficacy, anxiety and behavioral intentions relating to the combination of TRAK with standard care [28].

## Patient interviews

### Theme 1 – Experiences of the blended approach of standard ACL rehabilitation with TRAK

Using TRAK blended with standard physiotherapy enhanced the patient experience for most participants. The blended combination of their face-to-face care and the use of the website to support self-management gave patients access to the resources they needed at all times.

P13: There are certain things that I couldn’t remember from week to week but it was good to be able to go back and look at it again.

P8: Oh definitely, I couldn’t imagine it (rehab) without it. It is such a lot of work … it’s been 6 months since my surgery. You have to put in so much work. You need that support to keep.

P10: I mean…I do refer to internet anyway because of the accessibility issue. It’s so easy. So I think there are positives for that reason alone. It’s so easy to get to. If there is detailed information on there, I want to use it. I think it’s a good thing.

Patients found that the group structure was a positive experience. They discussed the learning environment and the support they felt from one another. They also described replacing something of the loss of their usual activities while they rehabilitated.

P2: I wasn’t sure how it would be to be part of a class but I really like it. I know it helps with volume control but actually it’s really nice to be around other people who have gone through the same thing and see the different stages before and ahead and how far you have come. How people are doing. And … I also think it’s a pretty good job of providing one to one during those classes too.

P9: One thing I really like was to come here and see the guys who had the same injury I had and you ask them, hey mate, what happened to you and they say football. Me too!

P6: Motivation and comparing yourself. It’s important to review other people and their training and it tells you a lot about what you should be doing. Why are you so good at that and why are you doing that so much better than what I do? You know it drives you. I had a very joyful moment of three times ten at 80 kg and others from my level couldn’t do it. I felt proud.

Eight patients mentioned that they felt lucky to have been in an area where ACL injury and surgery are commonplace and so an evidence-based rehabilitation group is provided with high levels of support. Their perception was that NHS care was varied and they felt lucky to be in this particular hospital.

P14: I felt very lucky to have happened to live in the right area.

P5: I think it’s really good because I have heard that other hospitals …doesn’t have a programme that is as good as yours and physios that look after you.

P6: I feel like I am one of those who won the postcode lottery.

P7: I think it has been pretty brilliant, the support I have received in comparison to other people I know who have gone to other hospitals.

### Theme 2 - Personal characteristics and rehabilitation engagement

Patients broadly seemed to find the ACL rehabilitation experience exceeded their expectations, but the commitment to rehabilitation was undoubtedly a burden for many patients and marked their lives in a significant way. Key concepts that recurred for patients were, commitment, motivation and confidence in physical ability.

### Commitment

P7: There is a lot to do …you have work and your life and then this rehab is a whole other thing.

### Motivation

P2: I think the toughest thing now is to keep the motivation to go to the gym three or four times a week. … you need to think of it as a lifestyle change, to keep fit…. Just trying to fit it with work and commuting and everything is hard.

### Confidence in physical ability

P7: I felt like I couldn’t do (things) and was letting people down. A lot of those jobs were hinging on me being able to do physical things. … so yes it has really affected me quite a lot.

Many patients described a loss of function and participation after ACL injury. There was a sense of being changed and often a feeling that the extent of their loss of function was not understood.

P6: I tried skiing without my ACL, I tried and tried but it felt awful. The more I thought about it, I needed the surgery. It would have ended up being injured more if I continued on that knee.

P9: I was in pain… I had 1 or 2 times …where I took a step and my knee just collapsed.

Some people reflected on how their pre-existing personality traits affected the way they engaged with both traditional physiotherapy and TRAK. Those who were disposed to improve self-care with the use of technology did so, whereas those who saw themselves as being differently motivated or skilled, engaged differently.

P14: In terms of tracking on the website I haven’t done it that well and I have been trying to work out why …and I am not very good at being consistent with my methods in anything in life and it includes that.

P1: I am lazy with the internet and it’s easier to watch a video and just do it but I’ll not report or log.

### Theme 3 – Attitudes to the internet and healthcare

As there are so many websites providing healthcare advice and services, patients expressed concern about knowing with confidence what information is ‘safe’ when it comes to digital resources. They worried about self-diagnosis, poor exercise technique and inappropriate advice exercise apps or websites where their personal injury and abilities were not fully understood and taken into account. Patients were very clear that they go online for information despite the risks and that they feel the NHS should be providing them with approved digital rehabilitation tools and information sources.

P5: it’s overwhelming too. You might think there is something wrong with you and you might do the wrong thing. You need to check with a real professional.

P14: Even if you think of putting that on YouTube with every other videos people will all go to the one that is NHS approved. Obviously that’s the one people would pick.

P6: If I don’t know how to do an exercise or something and I will go on YouTube or whatever but you get a varied standard and some may be downright dangerous. It is not good, for obvious reasons. So I think opt have a site that I know my physios or the hospital I am with, the NHS, has approved that these are the good stuff to do or links to the right stuff. That is kind of important.

### Theme 4 – Benefits of using TRAK for rehabilitation

The patient participants gave positive patient feedback on the key functions of TRAK. They felt that all the key functions (video-based personal plans, information, exercise logs and email support) impacted directly on their knowledge, motivation and confidence, but to differing degrees.

Patients felt that they went to the videos to inform their technique and give them confidence that they were doing the exercises correctly. They also reported that the videos reminded them of what exercises they should be doing.

P10: You can’t rate it highly enough really. It’s somewhere to go and check on what you’re supposed to be doing and make sure to do it right.

There was a mixed response to the exercise log, it was popular as a method to monitor progress but it was reported to be somewhat user-unfriendly and did not provide an option of a general progress report.

P6: It really makes me because I think, oh god I haven’t filled anything in to my log this week and it looks so bad. It really drags me to the gym, I look at it and think, I have to do something. I have to do something … even when I feel yuk.

P8: Again at the beginning I was inputting few things but it was adding half an hour onto my gym session. Trying to get a connection and all that.

The information section was popular immediately post-operatively and at phase-to-phase transitional points. Several patients suggested that the information was too wordy and would be improved by presenting it in video format and linking it with the exercise phases, i.e. a more seamless transition between the personal plans and information.

P6: In the beginning when you have more issues and you are fresh out of surgery and a bit ahhhhh. Then it’s very useful.

The ‘contact us’ section was not as popular as expected. Patients reported that they wanted the face-to-face reassurance when they had concerns. This identified a challenge for new technology to find effective ways of reassuring patients through a digital medium.

P10: I thought before I started using it that I would use the contact us more but didn’t because I waited until the session and asked my questions there. Maybe I didn’t come into such huge problems where I had to contact you urgently.

Some individuals emphasized the importance of TRAK to support rehabilitation when they were unable to attend physiotherapy due to work commitments or because of personal circumstances.

P14: I think the videos are like a revolution! It’s amazing …but it’s not like what you see on YouTube because you trust it. … it doesn’t exist anywhere else as far as I can see.

P3: There are lots of exercises that people can do. And if it’s too easy for you, you can move up different stages. Some harder ones and some easier ones… I think it gives you a focus and a way to approach it. It’s like a weekly target to do it and not think of the long road.

It also acted as a motivator to comply with their rehabilitation:

P6: Well TRAK works very well for me. Firstly, it makes me go to the gym. It really makes me because I think, oh god I haven’t filled anything in to my log this week and it looks so bad. I love the videos; I look at them every time before I do my exercise. Because I am paranoid about bad technique and sometimes so paranoid about it that I stay away from doing things.

### Theme 5 – Limitations of TRAK for rehabilitation

Patients were asked about their user experience of TRAK as a tool to support self-management. The answers were broadly divided into extrinsic and intrinsic factors. Patients discussed extrinsic problems they experienced, which could have been addressed by better physiotherapist management of the personal plans or e-mail links.

P4: in terms of communication. Right from the off, I write a couple of messages in the contact us section and I never received anything back. It kind of killed the point of the website for me... to a degree.

P10: I refer back to the paper in the class sometimes as that is more up to date than my plan….

Other extrinsic factors such as access to and the speed of Internet connection also affected usability. Without exception, every patient mentioned this as a limitation.

P15: The problem is the internet connection, in the gym it’s impossible to navigate between pages so I just gave up. Some apps you can use offline, that is what is needed.

P8: Again at the beginning I was inputting few things but it was adding half an hour onto my gym session. Trying to get a connection and all that.

Intrinsic factors referred to the website function itself. Patients discussed functional limitations of TRAK in comparison to other apps they were exposed to through the commercial market.

P9: (I used it) out of commitment (to the study). If it was more designy (sic) or there was an app easy to use then definitely…. If it was done like the Nike one, you can bet there would have been so many downloads…. There is nothing on there to say ‘come on’. Like the Nike app, if you run there is this voice saying ‘great job, you did 5 K today’.

P14: I found it difficult, well, clunky to add it add the bottom of every page. To put that in each time. It’s the kind of ins and out of the techy stuff … it wasn’t smooth.

### Theme 6 – TRAK in the future: How to increase the impact on positive behaviors

Patients saw the use of TRAK blended together with face-to-face physiotherapy as the future of ACL rehabilitation. They agreed universally that a digital exercise intervention was needed and they used their own experience with new technology to provide more information about their user needs. They would like to see the usability of existing functions improved, e.g. the ways of providing health-related information (educational videos alongside written information), easier identification of exercises (thumbnails alongside their descriptions), easier logging of exercise progression (slider scales and speech notes as an alternative to text input). New functionality that TRAK could benefit from mainly focused on personalization aspects such as prompts and cues that could be set individually, summary of overall progress, web chat, etc. The majority of patients felt that the web site needed to be converted into an app accessible on handheld devices such as tablets and smartphones and in particular with functions that are accessible offline.

P4: I would very much like to stress that it should be an app. It’s just that it would really help because it is really tricky on the phone. It’s hard in the gym I want to look at the examples really quick and remind myself …an app would be better. You can use it offline.

P9: I want it to prompt me, I want it to give me a weekly record, I want it to shout at me through the phone that I need to be doing my single leg squats today, that kind of thing. Something that comes up saying ‘don't forget this, do this today’.

They requested further prompts to emphasize exercises they may be missing, prompt a help link, a chat log that you could refer back to, a dashboard of completed sessions and milestones that can generate a weekly report, a set of different workouts that the can be automatically generated such as A, B & C, per week, a body map to show where they should feel the exercises, further links to relevant resources such as evidence or sport-specific information and pop ups that remind them where they are relative to milestones.

P6: I wish I could reflect all the exercise that I am doing. Even stuff that’s not on TRAK, so I can keep a complete record of my exercise and progress. Drills, squats that aren’t on TRAK. Sounds banal but there is a pride in showing all that I have done to myself and the physio. It’s what I do to get better.

P7: the kind of comparison I have is when I was going to do a half marathon and I used this app and it told me every day. Go and run this far, now do a strength and conditioning class and now go do some yoga.

Patients were very clear that they did not think TRAK was a replacement for physiotherapy, but should be used to complement face-to-face treatment in a ‘blended approach’. They were very keen to explain that it was an aid to their self-care when they were not in physiotherapy. Interestingly several patients who were in the middle stage of their rehabilitation did say that when they became too busy at times they were happy to manage through the website and attend physiotherapy less often.

P16: I would like to have both but I wouldn’t have it instead.

### Physiotherapist interviews

Physiotherapist discussed the benefit of having a website to enhance their patients’ self-management during rehabilitation. Physiotherapists explained that they want to improve their patients’ confidence, quality of exercise technique and compliance with exercise as the evidence suggests this would ensure better outcomes. The website provides a more evolved way of influencing these factors than traditional exercise handouts. They also noted that digital health options do not interest all patients and that patients’ self-selection is key to acceptability of digital interventions.

### Theme 1 - Benefits to physiotherapists

Physiotherapists expressed enthusiasm for TRAK on behalf of themselves and their patients, however they also seemed to acknowledge that not all patients would be suitable for this digital tool.

PHY1: We are giving back that locus of control to the patient …. You pick a patient who is appropriate for it. I mean there are so many patients who you may have say 6 appointments with, the middle three might just be exercise progressions. Some patients would be more than happy with email review… well, you could save the patient time too.

PHY4: Anyone that wants. Self-selecting group and definitely. I am refusing to be ageist because I have 80-year-old patients who email me and who are very engaged with technology. Equally I have 30 years olds that don’t care or don’t get it.

Rotational physiotherapists particularly felt that they used the website to educate themselves. That it was a go to summary of the education for each stage and a library of exercise ideas that they could use to help patients.

PHY3: As a junior physio I found it really useful in terms of information and a quick go to resource for reviewing milestones and do’s and don’ts…. I quite relied on it and it fed me information. Especially band 5 physios who come into a trust new and it’s a great learning tool to help you.

PHY1: If we had much more digital contact with patients I would be very happy with that. We could be emailing patients and caring a lot remotely. Simple progressions and checking on exercises could save contact time, patients’ own time and money I guess.

### Theme 2 - Suggestions to improve the usability of TRAK for physiotherapists

Of particular concern to physiotherapists was the time it took to build personal exercise programs and maintain them as patients progressed. Practical suggestions that recurred in interviews included the use of tablets in class to update programs quickly, thumbnail images alongside the names of exercises so they could quickly see what each one was without having to click on it and load new pages. Another suggestion was an inbuilt spelling checker, as one dyslexic physiotherapist felt they were slow in adding instructions because this functionality was not available. Physiotherapists would also like to see the exercise section have several ‘bolted’ key exercises for each stage.

PHY1: It would be good to build a smoother understanding of how we use the TRAK in the sessions. Some patients expect to be updated, some don’t and then we don’t know… in the sessions, having TRAK open on iPad or something would work. So patients could almost go through it with you as you upgrade it.

One physiotherapist was concerned about the use of patients in the videos because their exercise technique may not be perfect, e.g. a hop that showed inadequate valgus control of the knee. This emphasized the need to upgrade some of the videos. Another physiotherapist was concerned that not enough variations of exercises were available, which meant some exercise videos differed from what the patients were taught in the group.

Importantly, they would like to see a weekly dashboard or a report generated on what the patients have done. This could be printed as a notes record and can be added to paper or digital notes kept in the department instead of duplicating an exercise compliance and progression record.

Physiotherapists discussed the risks associated with TRAK training and management for physiotherapists. They were concerned about delays in picking up patient contacts and felt an emphasis on red flags in the information or red flag pop up reminders might help to identify potentially vulnerable patients.

PHY4: I have a disclaimer about slow contacts on my email and the phone number of the department in case of problems …It needs to have an out of office function or a re-routing function for when therapists are off. The information section, it should have …a clear red flag lists, when to stop and exercise, FAQ’s etc. again to reduce anxiety and reassure.

Further suggestions for improvement included providing information on using videos in addition to written material. One physiotherapist suggested that in line with commercial apps, perhaps a celebrity or famous athlete who had a similar injury would be involved in the production of information videos. Patient-experience videos were also suggested as well as voiceover instructions to some of the videos to highlight key guidance on technique. Similar to the patients, the physiotherapists wanted to see prompts and pop ups that engaged patients and directed to different sections of information. They also felt that a group or board should be reviewing the content on an ongoing basis so that the evidence is up to date and exercises or information can be added as appropriate. Opinions were mixed about how much information about physiology or advanced rehabilitation knowledge patients would benefit from. However, it was agreed that links to approved information or research were a good addition.

## Discussion

This study found that patients generally accepted a TRAK, a digital self-management support tool that was personalized with their own ACL rehabilitation plan and saw it as a positive addition to be blended with standard care. Physiotherapists likewise engaged well with the website as a support tool for patients and found it helpful to work with patients through this interface. Of the 17 interviewed patients and 4 physiotherapists, all thought that the blended approach of standard care and the website, notwithstanding its ongoing technical improvement, was an improvement on standard care alone. Every interviewed patient thought that the website should be a choice available to all ACL rehabilitated patients on the NHS.

In the interviews, the emergence of social influence, expectations and anxieties regarding TRAK usage reflect the established paradigm for acceptability of new digital technology discussed by Venkatesh et al. [28]. Performance expectancy (the degree to which an individual believes that using the system will help them attain goals), effort expectancy (the degree of ease associated with using the technology), social influence (the degree to which an individual perceives that important others believe they should use) and facilitating conditions (the degree to which an individual believes the organizational and technical infrastructure exist to support use) are considered the key factors in determining acceptability. As well as self-efficacy, anxiety and behavioral intentions.

Social influences in the group were evident where patients described their engagement with one another as an incentive for using the TRAK website. More importantly, individuals reported that interest in TRAK was dependent on the engagement of their physiotherapist. They reported less use if their physiotherapists were slow to respond to e-mails or because personal plans were not updated in a timely manner. This establishes the physiotherapist as the necessary agent behind the blending of standard care with TRAK. Physiotherapists expressed awareness of this and highlighted a number of facilitating conditions that influenced their ability to deliver this. Clinical time was the main challenge for TRAK management, but they felt that through use of better hardware such as tablet devices and improved organization of TRAK data, they could improve efficiency and maintenance of personalized programs.

Performance and effort expectancy of TRAK found a gap in functionality between this prototype and participants’ experience of commercial health apps such as ‘the Nike App’. In particular, they wished to see TRAK as an app with offline functions. Physiotherapists wanted a function for prompts and reminders that could be set by patients depending on patient goals in order to facilitate further engagement, and incentives such as, ‘if you achieve these target, you may begin the running program’ [33]. Patients and physiotherapists reported that TRAK was easy to use and did not frustrate their effort expectancy, with the exception of maintaining the log, though this was also discussed as having a positive effect on behavior.

The Behavior Change Wheel model outlines the importance of understanding the patients’ physical, social and psychological sources of behavior in order to understand developing interventions for changing behavior [34]. This group of patients described their sense of loss, their goals and the burden of a long and physically challenging rehabilitation program. These contextual factors inform their feelings about the target behavior, the rehabilitation program. The patients discussed how the website informed, motivated and improved confidence in carrying out the desired behaviors. Specifically the website functions provided clear plans, videos and instructions as well as persuading them with targeted information and an opportunity to record and monitor progress [34]. Overall, individuals indicated that TRAK helped them as part of a blended approach to physiotherapy (face-to-face and digital) as opposed to exclusive face-to-face or digital options.

Some patients who participated highlighted that they would not have chosen ordinarily to use a digital health intervention as they were not sure they would like it or it would be beneficial. Interestingly they worried about how their lack of usage would impact on the physiotherapists’ opinion of them. They described themselves as ‘lazy’ and ‘sorry’ [23]. This raises questions about how organizational changes to incorporate digital tools may affect patients who choose not to use these tools [28, 35]. Morden et al. found that “care must be taken to balance the needs of clinicians and patients whilst avoiding the scenario where patient information becomes a substitute rather than a supplement” [36]. Every patient that was interviewed without exception said that they did not see TRAK as an alternative to their face-to-face physiotherapy appointments but it should be used in a blended approach combining the digital tool with face-to-face. This is an approach taken by Bossen et al. with knee OA [20]. While some patients will continue to prefer standard care, this blended approach facilitates patients who wish to work more independently to do so safely. Some patients did say they would accept TRAK instead of appointments in stage 3 of their rehabilitation where exercise goals were clear and they felt confident to work independently with longer gaps between appointments. This can be explored further in a planned feasibility trial of TRAK for ACL patients [37] . Given the established psychological factors in determining return to function and sport the role of patient confidence in the blended approach is key to its usefulness [15]. As such TRAK is a unique digital tool for ACL reconstructed patients to facilitate the self-care component of their rehabilitation.

## Conclusion

Exploring patients’ opinions showed an evolved understanding of the potential benefits of a blended approach. TRAK aims to influence patient engagement and behavior change in line with established theory by motivating patients, giving them the capability to perform well and creating opportunities to record development toward well-defined goals. The study results suggest that TRAK, subject to technical improvement, was acceptable to patients in effort and performance expectancy. The value of TRAK was understood by participants to be a personalized reflection of their rehabilitation program with interactive components that aimed to inform, motivate and engage. However, physiotherapists highlighted organizational changes are needed to better integrate its use into standard physiotherapy practice.

## Abbreviations

ACL: Anterior Cruciate Ligament
NHS: National Health Service
PI: Principal Investigator
TRAK: Taxonomy for the Rehabilitation of Knee Conditions
